# A Practical and Theoretical Treatise on the Diagnosis, Pathology, and Treatment of Diseases of the Skin, Arranged According to a Natural System of Classification, and Preceded by an Outline of the Anatomy and Physiology of the Skin

**Published:** 1843-05-13

**Authors:** 


### BIBLIOGRAPHICAL NOTICE.

*A Practical and Theoretical Treatise on the Diagnosis, Pathology, and Treatment of Diseases of the Skin, Arranged according to a Natural System of Classification, and preceded by an outline of the Anatomy and Physiology of the Skin.* By Erasmus Wilson, Lecturer on Anatomy and Physiology, &c. &c. Philadelphia: Lea & Blanchard. 1843. 8vo. pp. 370.

The pathology of cutaneous diseases, which has so long occupied the French, has recently again attracted the attention of the English, after years of almost entire neglect. The land of Willan and Bateman seemed, until very recently, almost forbidden ground to the Dermatologist.

The earliest classification of diseases of the skin was the very imperfect one of situation. Plenck, of Buda, first classified cutaneous affections from the character of the eruption; and Willan extended and completed the idea in his celebrated nosological arrangement, which has been, with slight modifications, almost universally adopted since, both in Great Britain and in France. The Willanean classification, from its seizing on some one prominent feature as a distinctive character of the disease, bore a striking analogy to the Linnean Classification of the Vegetable Kingdom, and was hence called *artificial.* As by the one a tyro in botany may at once recognise a flower when in full bloom, so by the other the student may readily classify a skin-affection when at its period of full development; but, as in the one case, when the corrolla are yet unexpanded or the stamina have disappeared, the young botanist may be at fault with the Linnean system as his only reliance, so with the youthful Dermatologist, who, in the rise or decline of a disease, will find Willan a very deceptive and uncertain guide. Reflecting on the advantages and disadvantages of the Willanean Classification, and comparing it with the botanical systems in use, the idea struck Mr. Wilson, as he tells us, "that the study of diseases of the skin might be much simplified, and consequently facilitated, by the creation of a system which should embrace all the advantages offered by the Natural System, while it retained the benefits derivable from the Artificial System. It was this thought which gave origin to the system which I have endeavoured to illustrate in the following pages, and for which I have assumed the appellation of—Natural System of Diseases of the Skin." Mr. Wilson does not seem to be aware that 'this self same thought' entered, too, the brain of a Gallic confrére at a period somewhat antecedent. Dr. Charles Martins, Adjunct Professor of the Faculty of Medicine at Paris, ustained this view in a thesis, subsequently published in the Revue Medicale, Tome IV, 1835, entitled "*Les Principes de la Méthode Naturelle appliqués à la classification des maladies de la peau.*"

There is no class of affections in which a classification is so essentially necessary as that of the diseases of the skin. By it the labours of the student are materially abridged, and his inquiries greatly facilitated. Mr. Wilson proceeds in rather a tumid and figurative manner to eulogise this product of a lucky conception, and though his style is not very persuasive or convincing, an examination of his classification satisfies one of its "strength, its simplicity, its easy application, and its truth." The basis of the Natural System of Classification is Anatomy and Physiology. The dermis and its dependencies, its glands and follicles being the seat of all the affections of the skin, Mr. Wilson makes four Primary Divisions, as follows:

I. Diseases of the Dermis.
II. Diseases of the Sudoriparous Glands.
III. Diseases of the Sebaceous Glands.
IV. Diseases of the Hair and Hair Follicles.

The secondary divisions are made with reference to the complex nature of the tissues, or to the perversion of their functions.

The work itself is a very excellent digest of the present state of our knowledge on the diseases of the skin. We do not know that higher pretensions are claimed for it by its author. The student will find it a good manual, and the young practitioner a safe guide.

